# Dynamic MRI with Locally Low-Rank Subspace Constraint: Towards 1-Second Temporal Resolution Aided by Deep Learning

**DOI:** 10.21203/rs.3.rs-5448452/v1

**Published:** 2025-02-27

**Authors:** Eddy Solomon, Jonghyun Bae, Linda Moy, Laura Heacock, Li Feng, Sungheon Gene Kim

**Affiliations:** 1 Department of Radiology, Weill Cornell Medical College, New York, NY, United States.; 2 Center for Advanced Imaging Innovation and Research (CAI2R), Department of Radiology, New York University, New York, NY, United States.

## Abstract

MRI is the most effective method for screening high-risk breast cancer patients. While current exams primarily rely on the qualitative evaluation of morphological features before and after contrast administration and less on contrast kinetic information, the latest developments in acquisition protocols aim to combine both. However, balancing between spatial and temporal resolution poses a significant challenge in dynamic MRI. Here, we propose a radial MRI reconstruction framework for Dynamic Contrast Enhanced (DCE) imaging, which offers a joint solution to existing spatial and temporal MRI limitations. It leverages a locally low-rank (LLR) subspace model to represent spatially localized dynamics based on tissue information. Our framework demonstrated substantial improvement in CNR, noise reduction and enables a flexible temporal resolution, ranging from a few seconds to 1-second, aided by a neural network, resulting in images with reduced undersampling penalties. Finally, our reconstruction framework also shows potential benefits for head and neck, and brain MRI applications, making it a viable alternative for a range of DCE-MRI exams.

## Main:

Breast cancer is the most common cancer worldwide and is the second most common cause of cancer death for women in the United States^1^ with a lifetime risk of 13%^2^. In recent years, Dynamic Contrast Enhanced (DCE) MRI has been applied widely in high-risk screening population, resulting in sensitivity of 90–93%, compared with 48–63% for mammography and ultrasound combined^3^. DCE-MRI evaluates the vascular environment following the intravenous injection of a contrast agent, with signal enhancement characterized using quantitative kinetic information^4,5^. While the latest trend in acquisition protocols involves incorporating both detailed morphological information from high spatial resolution images and kinetic information^6^, most DCE-MRI exams solely rely on the qualitative comparison of morphological information before and after contrast. One reason for this is the poor temporal resolution available with current standard protocols, typically 60–120 seconds per volume, which severely obscures the fast-changing kinetics and the underlying physiological features of malignancy. The development of new acceleration strategies, on the order of a few seconds per frame, can potentially enable not only the high spatial resolution needed to detect small pathology^7^ but also accurate kinetic measurement^8^.

Balancing between temporal and spatial resolution poses a significant challenge in real-time dynamic MRI, where spatial resolution is often compromised for higher frame rates^9^. To address this challenge, a fast acquisition technique that combines compressed sensing^10,11^ and parallel imaging with golden-angle radial sampling, called Golden-angle RAdial Sparse Parallel (GRASP) MRI, has been developed to achieve high spatial and flexible temporal resolution^12,13^. The use of parallel imaging^14^ for accelerating MRI is widely used in the clinic today and is capable of generating images from undersampled measurements. However, its efficiency in breast DCE-MRI is often limited by the coil array design. Due to DCE-MRI’s sparse representation, compressed sensing^10,11^ has also become an attractive method by imposing suitable sparsifying transform along the temporal domain^15,16^. Another way to accelerate data acquisition while exploiting the dynamic correlations in dynamic MRI is to leverage its low-rank property^17^. Similar to compressed sensing, low-rank methods provide sparse representations of images over time by assuming they can be represented by a linear combination of a small number of principal components (PC), thereby allowing the recovery of dynamic images from sparse samples^18^. Low-rank approaches have been used in multiple applications, including quantitative parameter mapping^19,20^, MR fingerprinting^21,22^ and DCE-MRI^23,24,25^. With the assumption that all image voxels lie in the same temporal subspace and thus are globally correlated, the use of globally low-rank (GLR) modeling for basis function estimation has proven to be useful in representing global contrast change^17^. To further improve its effectiveness to capture more localized spatially signal dynamics, locally low-rank (LLR) modeling^26,27^ confines this correlation to local voxel neighborhoods by forming a spatially localized Casorati matrix. Lately, the utilization of deep learning (DL) networks has been shown to be effective in further accelerating MRI measurements by facilitating the recovery of highly undersampled data, making it especially useful in fast dynamic MRI^28,29^. However, the use of DL to accurately resolve DCE experiments under fast undersampled measurements has not yet been fully addressed.

In this study, we propose a new image reconstruction framework for radial k-space data, referred to as Enhanced Locally low-rank Imaging for Tissue contrast Enhancement (ELITE), which offers a joint solution to the aforementioned spatial and temporal DCE-MRI limitations. Our framework leverages a locally low-rank (LLR) subspace model to represent spatially localized dynamics based on tissue segmented information. By enforcing an explicit LLR subspace constraint of each breast tissue and learning its temporal basis from low-resolution undersampled radial DCE data, a subspace representation is generated and is used for the reconstruction of high-resolution images. Our framework also enables to generate dynamic image-series with flexible temporal resolution, up to 1-second, while exhibiting images with reduced undersampling penalty. For this purpose, a dedicated Residual Network (ResNet) was designed to effectively remove common streak artifacts at a high acceleration rate, while maintaining high temporal fidelity. Notably, our network was trained and tested on a unique publicly available dataset of raw k-space data recently generated and released by our group^30,31^. The proposed framework was evaluated for its image quality in comparison to other conventional reconstruction methods, and for its temporal information through a pharmacokinetic analysis. To the best of our knowledge, this is the first work where clinical breast DCE data acquired with radial acquisition is integrated with LLR subspace modeling and deep-learning network to achieve tissue-localized dynamics with high temporal resolution.

## Results:

### Enhanced locally low-rank (LLR) reconstruction for DCE-MRI

**Fig. 1:**
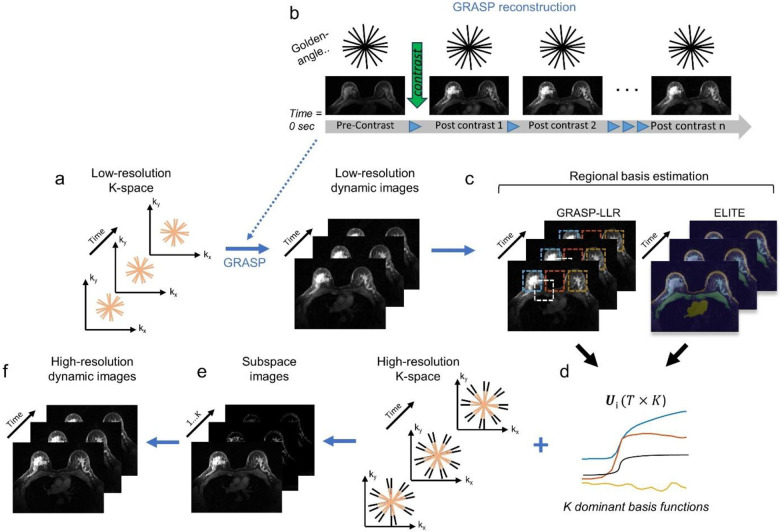
GRASP-based LLR reconstruction framework. (a-b) GRASP low-resolution images are reconstructed by solving the optimization problem in Eq 1. (c) Based on these images, temporal basis estimation is performed using a principal component analysis (PCA) for each block (‘GRASP-LLR’) or tissue-segments (‘ELITE’). (d) By utilizing the most dominant basis components, (e) high-resolution image-series is compressed to a low-dimensional subspace image-series. (f) High-resolution time-series is reconstructed by multiplying the set of coefficients by the basis functions and by solving the optimization problem in Eq 2.

A comparison between the GLR and LLR approaches for the use of DCE-MRI can be found in the Methods section (Supplementary Fig. 1). Following GRASP-based LLR approach, we propose the following reconstruction framework (Fig. 1). This framework takes advantage of the radial sampling trajectory, where the densely sampled k-space center (Fig. 1a, pink radial spokes) allows a reliable reconstruction of low spatial resolution dynamic image-series with standard GRASP reconstruction (Fig. 1b). Based on these low-resolution dynamic images, LLR basis functions are estimated using principal-component-analysis (PCA) for n number of blocks (‘LLR’) or tissue-segments (‘ELITE’) (Fig. 1c), each represented by K most dominant principal components (PC) (Fig. 1d). Next, the high-resolution image-series is compressed to a low-dimensional subspace (Fig. 1e), and as a final step, the high-resolution time-series is reconstructed by multiplying the set of coefficients by the basis functions (Fig. 1f).

### Improved lesion delineation and noise reduction

**Fig. 2:**
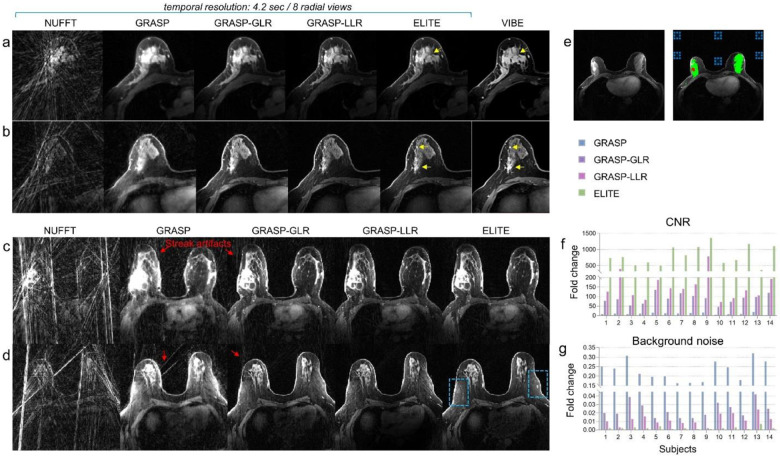
Lesion delineation enhancement and noise reduction. (a-b) Two representative cases of tumor lesion reconstructed by GRASP-LLR and ELITE methods, after contrast injection. Conventional VIBE images based on Cartesian sampling were limited by temporal resolution of 120 sec, while GRASP reconstruction methods provide higher temporal resolution of 4.2 sec (8 spokes) in these examples. Malignant and fibroglandular tissues are indicated by yellow arrows. (c-d) Two representative cases showing the reduction in background streak artifacts achieved by GRASP-LLR and ELITE reconstruction methods, compared to GRASP and GRASP-GLR exhibiting strong streak artifact and blurry anatomy. In this example, our framework (‘ELITE’) also exhibits clearer anatomical details (blue dotted frames). (e) Contrast to Noise Ratio (CNR) was calculated between the malignant lesion and fibroglandular tissue (red and green ROI’s, respectively) and noise level was calculated as the standard deviation of six background ROI’s (dotted blue squares). Fold changes in (f) CNR and (g) background noise, relative to NUFFT, are shown for GRASP, GRASP-GLR, GRASP-LLR and ELITE.

The GRASP methods were utilized for the reconstruction of breast images with a temporal resolution of 4.2 sec (Fig. 2). The proposed reconstruction framework (Fig. 1), based on either blocks (‘GRASP-LLR’) or tissue-segments (‘ELITE’), showed improved delineation of fine tissue structure (Fig. 2a and 2b) compared to conventional NUFFT, GRASP, or GRASP-GLR methods. While the contrast between the tumor lesion and surrounding tissue appears noisy and blurry for the latter methods, a sharp delineation of the lesion is achieved with both GRASP-LLR and ELITE methods. Moreover, while conventional post-contrast VIBE images, based on Cartesian acquisition, were acquired with a temporal resolution of 120 seconds, GRASP-LLR and ELITE images exhibited comparable image quality but with a temporal resolution of 4.2 seconds. Using GRASP-based LLR approach, we can also gain a substantial reduction in background noise by representing the background signal with a single principal component (K=1). While NUFFT, GRASP and GRASP-GLR methods experience stronger streak artifacts (Fig. 2c and 2d), our framework (‘ELITE’) demonstrated a reduction in background noise, revealing sharper breast vascular properties (Fig. 2d, indicated by blue dotted frames). Video 1 and 2 (in the supplementary) exhibit the streak artifacts time-series observed in each of GRASP reconstruction methods. The advantage of ELITE framework is also evident in other breast cases, revealing consistent sharper vascularity and clear breast morphological features (supplementary Fig. 2, indicated by yellow arrows). These features are largely obscured by streak artifacts when reconstructed by NUFFT, GRASP, GRASP-GLR and GRASP-LLR methods (supplementary Fig. 2, indicated by red arrows). A quantitative analysis of contrast-to-noise ratio (CNR) (Fig. 2f) and background noise estimation (Fig. 2g) was conducted across multiple breast cancer cases by taking into consideration the fibroglandular tissue (Fig. 2e, green mask), the lesion (Fig. 2e, red mask) and its background signal (Fig. 2e, dotted blue squares). All GRASP methods showed a fold increase in CNR and a fold decrease in background noise levels compared to NUFFT with 10.4±4.5/4.6±1.1, 88.9±25.3/47.1±16.4, 194.9±187.6/134±151.4 and 807±298.8/529.8±248.2, for GRASP, GRASP-GLR, GRASP-LLR and ELITE, respectively. Additionally, a significant increase in CNR was found between GRASP methods (P<0.001), as well as a significant decrease in noise level (P<0.001), with ELITE achieving the highest scores in both categories.

### Breast DCE with high temporal fidelity

**Fig. 3:**
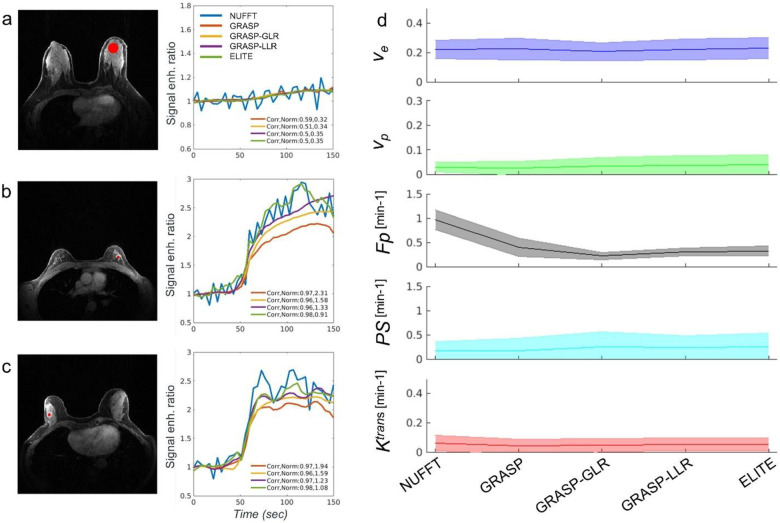
GRASP high temporal fidelity. Signal enhancement ratio curves of (a) fibroglandular tissue, (b) benign and (c) malignant lesion tissue, are plotted for different GRASP reconstruction methods. Each curve is scored for its correlation and Euclidean norm, in comparison to NUFFT. All curves are plotted based on the average signal given by region of interest (ROI) marked in red. (d) Pharmacokinetic (PK) parameters ($V_{e},V_{p},Fp,PS,K^{trans}$) of the two-compartment exchange model were measured for healthy fibroglandular tissue cases (n=21), showing comparable parameter values across reconstruction methods.

To investigate how reliably GRASP reconstruction methods can represent temporal information, we assessed their temporal fidelity information through the signal enhancement-based tissue over time and pharmacokinetic model (PKM) analysis. Signal enhancement sampled from fibroglandular tissue (Fig. 3a) did not show major differences between GRASP, GRASP-GLR, GRASP-LLR and ELITE, when compared to that of NUFFT (Pearson correlation coefficient and Euclidean norm 0.59/0.32, 0.51/0.34, 0.5/0.35, 0.5/0.35, respectively). Signal enhancement based on ELITE, exhibited a slightly higher correlation and lower norm values for benign (0.98/0.91, Fig. 3b) and malignant lesion tissue (0.98/1.08, Fig. 3c), among the rest of the GRASP methods. The mean and standard deviation of PKM parameters were estimated for fibroglandular tissues and compared across GRASP methods (Fig. 3d). Except for blood flow (${\text{F}}_{\text{p}}$) that was found to be significantly higher (P<0.001) for NUFFT than for the GRASP methods, all other pharmacokinetic parameters exhibited consistent values with no significant difference across all five reconstruction methods. Pharmacokinetic parameter maps of $v_{e},v_{p},F_{p}$ and $K^{trans}$ for fibroglandular tissue, benign and malignant lesion (Supplementary Fig. 3–5, respectively), corresponding to those shown in Fig. 3a–c, demonstrated comparable parametric maps, except for NUFFT that was distorted by streak artifacts.

### Fast DCE-MRI aided by deep learning network

**Fig. 4:**
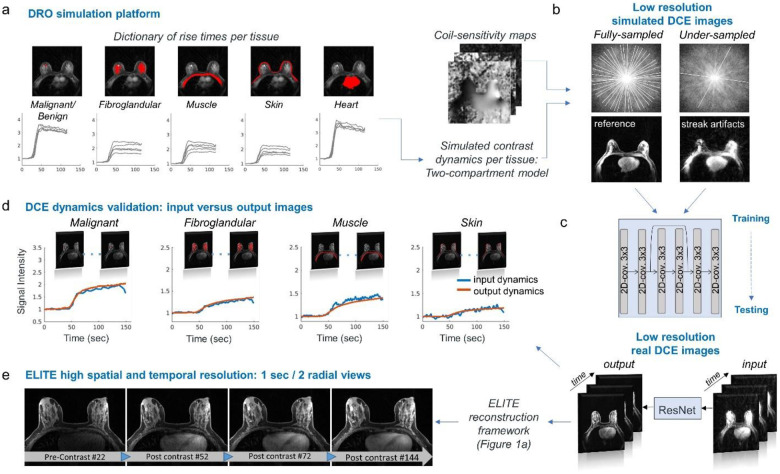
ELITE reconstruction aided by Residual Neural Network (ResNet). (a) A ResNet was trained with simulated data generated by a Digital Reference Object (DRO) platform. To augment training data, each tissue was estimated for its rise time, creating a dictionary of typical rise times per tissue segment. (b) DRO-simulated data included undersampled and fully sampled low-resolution DCE images. (c) The trained ResNet was tested on real undersampled DCE images, yielding images with reduced noise. (d) ResNet ‘input dynamics’ and ‘output dynamics’ image-series yielded temporal information with high correlation for different breast tissues. (e) Output image-series generated by ResNet, were plugged into ELITE reconstruction framework for basis estimation (Fig. 1a), yielding high spatial and temporal resolution ELITE images-series.

Building on the image quality and temporal fidelity achieved with our proposed framework, we next utilized it for generating fast dynamic data with 1-sec temporal resolution (2 spokes/frame). As expected, the reconstruction of these undersampled images resulted in strong streak artifacts that distort all parts of breast tissue. To address this challenge, a Residual Neural Network (ResNet) was trained with simulated data, generated by a Digital Reference Object (DRO) platform^30^ (Fig. 4a). To enhance machine learning training augmentation, each tissue (i.e., malignant or benign, fibroglandular, pectoral muscle, skin and heart), across all available cases, was estimated for its rise time, creating a dictionary of typical rise times per tissue. Low resolution simulated DCE dynamic data included fully sampled images as reference and undersampled images reconstructed with two spokes (Fig. 4b). Once trained, ResNet was tested on real undersampled images (‘input), yielding images with reduced noise (‘output’) (Fig. 4c). A representative time-series data of the ResNet input and output can be found in Supplementary (Video 3). Furthermore, to validate the temporal fidelity of the data generated by ResNet, we compared the temporal information of the output image-series with the input image-series, plotted for each tissue separately (Fig. 4d). Dynamic curves showed high correlation and norm of 0.99/0.4, 0.99/1.08, 0.98/0.61, 0.92/0.38 for fibroglandular, malignant, muscle and skin tissues, respectively. Finally, the output image-series generated by ResNet, were plugged into our reconstruction framework (Fig. 1a) for bases estimation, yielding high spatial and 1-sec temporal resolution images-series (Fig. 4e) exhibiting 144-time frames.

### Clinical assessment through qualitative and quantitative DCE analysis

**Fig. 5:**
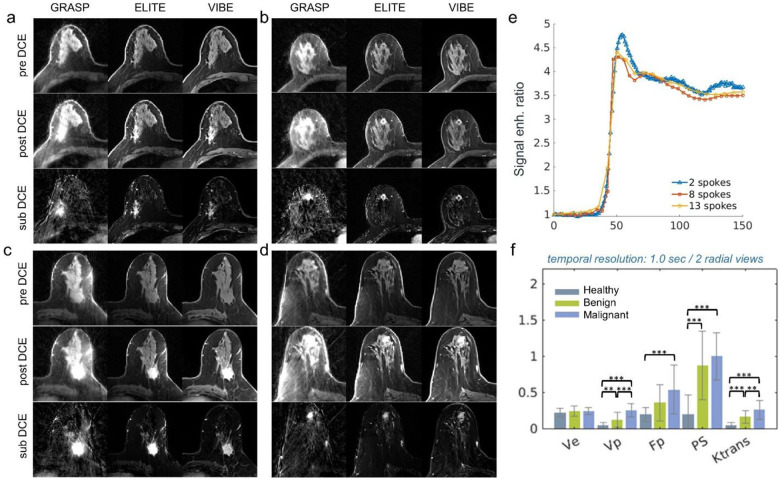
Qualitative assessment of DCE images and quantitative PKM analysis. (a-d) Qualitative assessment of DCE images of four representative malignant cases reconstructed with ELITE, with temporal resolution of 4.2 sec, displaying pre-, post- and sub- (subtraction) DCE images. (e) Sampling the DCE signal in the aorta based on ELITE images at different temporal resolutions of 1 sec (2 spokes), 4.2 sec (8 spokes) and 6.8 sec (13 spokes), showed a clear improvement in the rapid wash-in part for the high temporal resolution. (f) PKM analysis with ELITE images of 1-sec temporal resolution yielded good separation between healthy, benign and malignant cases. P-values were calculated by one way ANOVA (* P < 0.05, ** P < 0.01, *** P < 0.001). All images share the same dynamic scale.

For the purpose of assessing breast tissue under typical clinical conditions, we examined the use of our framework for the assessment of pre-, post-, and subtracted DCE (sub-DCE) images reconstructed with temporal resolution of 4.2 sec (Fig. 5a–d), and its use for quantitative pharmacokinetic analysis with a 1-sec temporal resolution (Fig. 5e–f). The pre-, post-, and sub-DCE images of ELITE demonstrated a clear improvement in image quality and lesion delineation compared to the conventional GRASP method. They also exhibited high correspondence with post-contrast conventional VIBE images. The advantage of our framework over conventional GRASP was also consistent across other examples (Supplementary Fig. 6–10). When sampling the aorta lumen based on ELITE images with different temporal resolutions (Fig. 5e), time resolution of 1-sec (2 spokes) exhibited a fast-rising curve with the highest peak compared to 4.2 sec (8 spokes) and 5.25 sec (13 spokes). Next, we examined the use of ELITE images for quantitative PKM assessment of healthy fibroglandular tissue, benign and malignant lesions. A PKM analysis (Fig. 5f), based on 1-sec temporal resolution, showed a clear separation between patient groups for most pharmacokinetic parametric parameters. While the mean values of extracellular-extravascular space volume fraction $\left(v_{e}\right)$ showed no significant difference between healthy, benign and malignant cases, the blood volume fraction $\left(v_{p}\right)$, blood flow $\left(F_{p}\right)$, permeability surface area product (PS) and exchange rate ($K^{trans}$), exhibited statistically significant differences between patient groups.

### Applications in imaging the brain and the neck

**Fig. 6:**
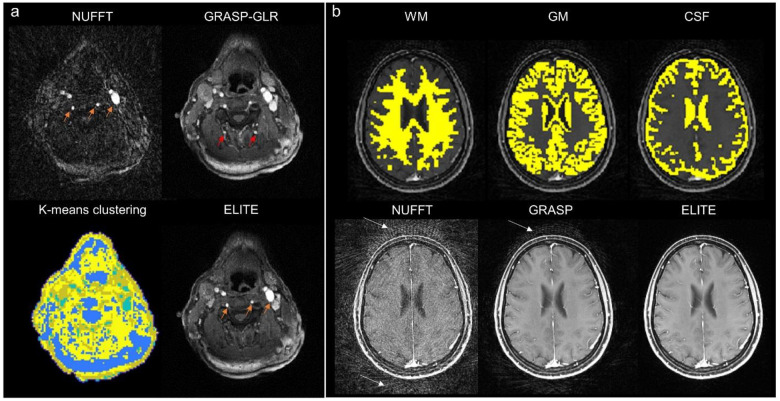
Applications in DCE-MRI of the head and neck and the brain. Other anatomical information can be utilized for ELITE image reconstruction: (a) ELITE DCE head and neck data yields time-series images with accurate temporal enhancement of arterial and venous blood, consistent with NUFFT (orange arrows), while GRASP-GLR exhibits a false enhancement in posterior vessels (red arrows). (b) Brain imaging can also be utilized for ELITE segmented-based reconstructed composed of white matter (WM), gray matter (GM) and cerebrospinal fluid (CSF) maps, resulting in enhanced T1-weighted anatomical images with reduced streak artifacts (white arrows).

Our reconstruction framework can also be utilized for other DCE-MRI applications. For head and neck imaging (Fig. 6a), a K-means clustering-based segmentation was used for ELITE reconstruction, yielding time-series images with accurate temporal enhancement of blood veins and carotid artery, consistent with NUFFT (Fig. 6a, orange arrows). However, for the same time point, GRASP-GLR showed false enhancement, as indicated by the red arrows. Another important anatomy that can benefit from our proposed framework is the brain, where white matter (WM), gray matter (GM) and cerebrospinal fluid (CSF) information can be utilized for its tissue-based reconstruction (Fig. 6b, upper panel), resulting in T1-weghted DCE-MRI images with lower noise artifacts, compared to conventional NUFFT and GRASP reconstruction (Fig. 6b, lower panel, white arrows).

## Discussion:

In this work, we propose a novel radial DCE-MRI reconstruction framework, tailored for clinical breast DCE-MRI exams, that leverages new acceleration concepts such as low-rank subspace modeling and machine learning, while providing sharp images in a single scan. Specifically, the proposed framework (‘ELITE’) is shown to not only improve breast DCE image quality in terms of CNR and noise reduction, but also provide reliable temporal information, even at 1-sec resolution. This work has the potential to improve current breast DCE-MRI protocols and may also benefit head and neck, and brain DCE-MRI.

Standard DCE-MRI imaging methods are limited by the need to balance between spatial resolution, temporal resolution, and scan time. As demonstrated in other patient exams^32,33^, radial GRASP imaging can serve reliably and is able to deliver comparable diagnostic performance to conventional DCE-MRI. Moreover, the possibility of radial imaging to flexibly undersample its data while providing diagnostic images, is an advantage over counterpart Cartesian protocols. However, for the purpose of DCE-MRI multiphase evaluation, the performance of standard GRASP is still limited by a relatively low temporal resolution (e.g., 15–20 s/image volume), which is prone to image blurring. The ability to reconstruct DCE-MRI time-series from limited data is due to the inherent undersampling nature and the high degree of sparsity of these experiments. Building upon previous work^17,20,24^, a reliable low-dimensional representation of temporal DCE-MRI information can be achieved with low-rank subspace modeling.

One way of estimating a temporal basis through low-rank subspace modeling is by a model-based approach^19,21^. By utilizing a physical signal model that generates an ensemble of possible signal enhancement patterns, a temporal basis can be generated using principal component analysis (PCA). However, a single physical model of a breast DCE-MRI may not be sufficient to accurately represent the dynamics of all breast tissues. Alternatively, a data-driven approach that estimates its temporal basis from real training data^34^, acquired separately or from low-resolution data, can be more reliable and efficient. In this work, temporal basis is estimated using low-resolution data, without the need for additional data acquisition. In this sense, the geometry of radial sampling provides an optimal solution, as undersampling rates are much lower around the central k-space than the periphery. Thus, low spatial resolution data can provide sufficient temporal information for estimating a temporal basis, which can then be utilized to represent the high-resolution image-series in a low-dimensional subspace^35^. To ensure that no breast anatomy is lost within the low-resolution images, the images in this work were restricted to half the original spatial resolution.

Moreover, given that DCE correlations tend to be higher among neighboring voxels, it is logical to confine the estimation of the temporal information to local voxel neighborhoods through locally low-rank (LLR) modeling. Indeed, as opposed to the globally low-rank (GLR) approach that estimates its temporal basis functions based on the whole image, we show that using GRASP with locally low-rank (LLR), either with block (‘GRASP-LLR’) or tissue segment-based reconstruction (‘ELITE’) (Supplementary Fig. 1), a sharper DCE time-series images can be generated with negligible streak artifacts (Fig. 2a–b and Supplementary Fig. 2), which results with higher CNR and lower background noise (Fig. 2f–g). Nevertheless, in some cases, GRASP-LLR still exhibited some residual streaking artifacts due to blocks located on the boundary between tissue and background signal (Supplementary Video 2). As such, block-based reconstruction requires some parameter tuning to balance between small size block, characterized by high sensitivity to nearby structures, and large size block, characterized by less specific correlated structures^36^. Notably, in our study, common block flickering^19^ across the time domain was barely noticeable, due to an overlapping sliding-window strategy used^37^.

Overall, employing the tissue segment-based reconstruction (‘ELITE’) approach, where each tissue type is represented by a set of principal components that capture the unique temporal dynamics for that tissue (Supplementary Fig. 1, far right), resulted in improved image quality. This approach was further extended to confine background noise using a single principal component, resulting in a significant reduction in background noise and streak artifacts. One reason for the sharper images by GRASP-LLR and ELITE over conventional GRASP images, is the ill-conditioning of the GRASP reconstruction problem^18^. For non-Cartesian imaging, such ill-conditioning often shows up as blurring artifacts, when the reconstruction has not yet converged. Notably, our reconstruction framework approach, shown in Equation 2, exhibited faster and more efficient convergence by fewer number of iterations (Supplementary Fig. 11), exhibiting 64% reduction in comparison to conventional GRASP, and 33% reduction in comparison to GRASP-GLR.

To test our reconstruction framework in providing reliable temporal information, we compared all GRASP reconstruction methods and their corresponding pharmacokinetic parameters (Fig. 3), as there is no established gold standard for measuring these parameters. Except for NUFFT that was heavily distorted by streak artifacts, all other reconstructions methods showed comparable parametric maps (Supplementary Fig. 3–5). Studies have shown that a temporal resolution of at least 16 seconds is necessary to depict tracer dynamics of tumor, and towards 1-second to capture the dynamics of arterial input function (AIF)^8^, both of which are key factors in the pharmacokinetic modeling. However, reaching higher temporal acceleration requires higher undersampling factors which are associated with Nyquist violation and thus result in stronger streak artifacts.

To address this challenge, we designed a dedicated Residual Neural Network (ResNet) with two main goals in mind: i) to effectively overcome the spatial undersampling penalty (i.e., streak artifacts), while ii) maintaining high temporal fidelity. Deep neural networks have demonstrated their effectiveness in reducing MRI artifacts and noise in non-Cartesian image artifact reduction^38^. However, their ability to mitigate undersampling artifacts in the presence of contrast injection while maintaining reliable temporal information has been limited. Moreover, the lack of large open-source databases for DCE-MRI has posed a significant hurdle for training and testing new network approaches. In this study, we used a new Digital Reference Object (DRO) toolkit, recently released by our group, to generate breast DCE data, undersampled and fully-sampled, which were then utilized for the training of our Residual Network (ResNet). Using ResNet, ELITE reconstruction was able to yield reliable 1-sec DCE time-series with high temporal fidelity, free from streak artifacts (Supplementary Video 3).

For optimal diagnostic assessment of breast DCE-MRI, both qualitative examination of pre-, post- and sub-DCE images, as well as quantitative kinetic analysis, should be used^6^. Since high temporal resolution is not required for qualitative morphology examination, ELITE images were reconstructed with 4.2 sec temporal resolution, resulting in clearer and sharper anatomy and good correspondence to conventional VIBE images (Fig. 5a–d). To quantify vascular properties through kinetic analysis, high temporal resolution is important. Therefore, ELITE images were reconstructed with 1-sec temporal resolution, exhibiting improved enhancement curves when sampled from the aorta (Fig. 5e), compared to lower temporal resolutions. The kinetic parameters estimated for fibroglandular tissue, benign and malignant lesions were within the range of other reported studies^39,40^, indicating a good separation between the three tissue types.

Several potential future directions can be considered for utilizing our framework in other DCE-MRI applications. For head and neck imaging (Fig. 6a), ELITE images showed an accurate temporal enhancement of blood in the veins and carotid artery, consistent with NUFFT (Fig. 6a, orange arrows). Furthermore, by performing temporal basis estimation for white matter (WM), gray matter (GM), and cerebrospinal fluid (CSF), we achieved improved brain images with minimal artifacts patterns (Fig. 6b, white arrows). Another interesting potential application of this framework is for the improvement of breast fat saturation (Supplementary Fig. 12). In this example, fat (masked in blue) was represented by a single principal component, effectively removing residual fat signals and improving overall breast image quality.

In conclusion, among other advance developments in MR breast cancer imaging such as diffusion^41^, hyperpolarization^42^ and spectroscopy^43^, DCE-MRI remains the most effective clinical method today. In this study, we present a valuable step towards establishing a new image reconstruction framework for radial imaging as a viable alternative for breast DCE-MRI exams. Overall, our approach offers enhanced image quality and high temporal fidelity, with potential impact in other DCE-MRI applications.

## Methods:

### Patient population

This study was conducted with fifty-five breast cancer patients who underwent clinical diagnostic breast MRI exams. It was performed with the approval from our Institutional Review Board and a written informed consent was obtained from each subject. It included patients with malignant lesions (n=24; 30–75 years old; mean 50.5 years) and patients with benign lesions (n=31; 25–68 years old; mean 40.5 years). Malignant lesions included invasive ductal carcinomas (IDC) (n=19), invasive lobular carcinomas (ILC) (n = 3), a mix of IDC and ILC (n=1), and ductal carcinoma in situ (DCIS) (n=1). The breast lesion tissue (malignant and benign) was manually segmented by a trained breast imaging radiologist (LH with 6 years of experience), while the breast healthy fibroglandular tissue was manually selected from contralateral benign breast cases.

### Data acquisition

Patients were scanned on a 3T MRI scanner (MAGNETOM TimTrio, Siemens Healthcare) with a 16-channel breast coil. Diagnostic exam included DCE-MRI with a free-breathing gradient-echo radial volume-interpolated breath-hold exam (VIBE) sequence and reconstructed with Golden-angle RAdial Sparse Parallel (GRASP)^13^. Scanning parameters included 288 radial-views, 192 slices with partial-Fourier, TE/TR=1.8/4.87ms, flip-angle=10 degrees, field of $\text{view}=320\times 320\times 212\;{\text{mm}}^{3}$, bandwidth = 520 Hz/pixel and spatial resolution of $1.0\times 1.0\times 1.1{\;\text{mm}}^{3}$. The GRASP DCE-MRI total scan time was 2.5 min. A single SPAIR (Spectrally selective Adiabatic Inversion Recovery) fat saturation pulse (116.6 ms) was applied once every golden angle view. A single dose of gadobutrol (Gadavist, Bayer Healthcare Pharmaceuticals) at 0.1 mmol/kg body weight was injected at 2 mL/s intravenously at the 30-seconds into the scan. In addition to radial VIBE scan, a Cartesian gradient-echo VIBE scan with fat-suppressed was used. Cartesian VIBE was acquired one phase before radial VIBE, and three phases post-contrast. Each T1-weighted VIBE scan took 120 seconds with TR/TE=1.52/4.01ms, flip-angle=12 degrees, and spatial resolution of $1.2\times 0.7\times 1.1{\;\text{mm}}^{3}$. VIBE images were used for image segmentation^30^.

### Globally versus locally low-rank reconstruction for DCE MRI

The globally low-rank (GLR) model enforces that dynamic image-series with a low-rank condition can be represented by a few dominant basis functions and their corresponding coefficients (Supplementary Fig. 1a). The GLR approach estimates its basis function from the whole image, which could be less effective in representing spatially localized signal dynamics commonly observed in breast DCE images. With locally low-rank (LLR) approach, the image can be divided either into blocks (LLR, Supplementary Fig. 1b) or tissue-segments (ELITE, Supplementary Fig. 1c). This way each region can be decomposed into a product of temporal basis functions. Following the ELITE reconstruction approach, each tissue (Supplementary Fig. 1c, far right) can be represented by its own set of singular values, which can then be expressed by K most dominant basis components.

### Image reconstruction

Our reconstruction framework (Fig. 1) can be either applied in a block-wise (‘GRASP-LLR’) or tissue-segments (‘ELITE’) approach. GRASP low-resolution (L×L) dynamic image-series (${\text{M}}_{L}$) are reconstructed (Fig. 1b) by solving the optimization problem:

[1] $$\underset{{\text{M}}_{L}}{\text{argmin}}\frac{1}{2}{\left\|{\text{y}}_{L}-E{\text{M}}_{L}\right\|}_{2}^{2}+\lambda {\left\|S{\text{M}}_{L}\right\|}_{1}$$

where ${\text{y}}_{L}$ is the low-resolution dynamic k-space data (L=160) re-gridded to a Cartesian grid using GROG^44^, which includes the corresponding weighting function (${\text{w}}_{L}$) to compensate for the radial sampling density. $E=\sqrt{{\text{w}}_{L}}\Phi F{\text{C}}_{L}$ is the low-resolution encoding operator incorporating coil sensitivities $\left({\text{C}}_{L}\right)$, Fourier transform (F) and undersampling operator (Φ).S is a sparsifying transform applied along the temporal dimension, such as the finite difference operation in this study to enforce total variation, with a weighting factor λ as the regularizer. After GRASP reconstruction, an LLR basis functions are estimated (Fig. 1c) from the low-resolution dynamic images using principal-component-analysis (PCA) for n number blocks (‘GRASP-LLR’) or tissue-segments (‘ELITE’). The dynamic image-series $\left({\text{M}}_{L}\right)$ is represented as ${\text{M}}_{L}=\sum_{i=1}^{n}U_{i}V_{ki}$ where $U_{i}$ is the temporal basis (T×K) of the i-th region and $V_{ki}$ represents the number of K associated coefficients (K×M) with M voxels in the i-th region. The number of principal components (PC), K, for ELITE reconstruction was empirically tested (Supplementary Fig. 13), where K=6 was found to be optimal in terms of lesion enhancement representation and the noise level. Increasing the number of PC’s increases background noise and may also result in less efficient compressed-sensing reconstruction, as already discussed elsewhere^19,23^. Following the basis estimation, the high-resolution images is compressed to a low-dimensional subspace image-series (Fig. 1e) and a final high-resolution time-series is reconstructed (Fig. 1f) by multiplying the set of coefficients by the basis functions and solving the optimization problem:

[2] $$\underset{V_{k}}{\text{argmin}}\frac{1}{2}{\left\|y-E\sum_{i=1}^{n}\;U_{i}V_{ki}\right\|}_{2}^{2}+\lambda {\left\|S\sum_{i=1}^{n}\;U_{i}V_{ki}\right\|}_{1}$$

where $y_{L}$ is the high-resolution dynamic k-space data (L=320) shifted to a Cartesian grid using GROG^44^. Block-wise reconstruction (‘GRASP-LLR’) was performed where each image was divided to 10×10 blocks, resulting in 32×32 block size for low-resolution images. For high-resolution image reconstruction, a block size resulted in 64×64. Tissue-segmented reconstruction (‘ELITE’) was composed of six regions (Fig. 1c): background, lesion (malignant or benign), fibroglandular, pectoral muscle, skin and heart. For each case, reconstruction was conducted with the slice with the largest lesion. For the head and neck DCE MRI data, K-means clustering was applied with four segments along the time domain. For brain DCE-MRI data, segmentation maps were generated for white matter (WM), gray matter (GM) and cerebrospinal fluid (CSF) maps using FSL^45^. GRASP dynamic images were reconstructed with 8 spokes per frame, yielding a temporal resolution of 4.2 sec/frame. To capture contrast dynamics with a high temporal resolution, ELITE dynamic images were reconstructed with 2 spokes per frame, yielding a temporal resolution of 1 sec/frame. The iterative reconstruction conducted in Eq. 1 and 2 used a nonlinear conjugate-gradient algorithm with seven iterations, repeated three times, and TV regularization weighting of λ=0.01. Number of iterations and λ value was empirically tested to balance between image quality and noise reduction (Supplementary Fig. 14). Reconstructions were performed on a Linux workstation with one 16-core CPU (Intel(R) Core (TM) i9-10980XE CPU @ 3.00GHz) and 256 GB memory.

### Network architecture and simulation

A 2D Residual Neural Network (ResNet) with ten convolutional layers was implemented in Pytorch and trained using the Adam optimizer. The network was trained in a supervised fashion using a ${\text{L}}_{2}$-norm loss function between undersampled images reconstructed with two spokes and fully sampled reference images. The training data was generated by a breast Digital Reference Object (DRO) simulation platform^30^ that creates realistic morphology and contrast kinetic of breast tissues. Training datasets for the simulation were derived from a bigger repository database that we recently released^31^. Training included eighteen cases (nine malignant and nine benign) and two cases for validation (one malignant and one benign). To enhance machine learning training augmentation, each tissue (i.e., malignant or benign, fibroglandular, pectoral muscle, skin and heart) across all 55 available cases, was estimated for its rise time (Fig. 4a), creating a dictionary of typical rise times per tissue. This information was then incorporated into our DRO simulation platform (Fig. 4b), generating 50 randomly augmented datasets per existing case. In total, this resulted in 900 different datasets for training and additional 100 datasets for validation.

### CNR and noise estimation

Contrast-to-noise ratio (CNR) estimation was performed between a malignant lesion (Fig. 2e, red region) and fibroglandular tissue (Fig. 2e, green region) segmentation regions $\left(CNR=\frac{|{\text{Mean}}_{\text{malignant}}-{\text{Mean}}_{\text{fibroglandular}}|}{\text{STDE}V_{\text{Noise}}}\right)$. To minimize the influence of streak artifacts on noise estimation $\left(\text{STDE}V_{\text{Noise}}\right)$, noise was calculated from the subtraction of consecutive dynamic images (after contrast injection), such that the only difference between the reconstruction methods is due to random noise^46,47^. Standard deviation of noise was calculated based on six ROI’s sampled in the background (Fig. 2e, dotted blue frames). CNR and background noise estimation were performed on fourteen malignant cases and was calculated for fold change between each GRASP reconstruction methods and conventional NUFFT (Non-Uniform Fast Fourier Transform).

### Dynamic signal and pharmacokinetic model analysis

Enhancement signal plots sampled for different tissues (Fig. 3, Supplementary Fig. 13) were evaluated in comparison to the dynamic signal derived from NUFFT images as a reference to assess temporal fidelity and as demonstrated in the original GRASP study^13^. To assess the dynamic temporal information of each GRASP reconstruction method, we determine the range of pharmacokinetic (PK) parameters using the two-compartment exchange model^4^. This model was used to include potential differences between the contrast concentration curves measured at an upstream artery and those in the capillary bed. The model adopts two compartments: the extracellular-extravascular space (EES) with the volume fraction of $v_{e}$ and the blood plasma compartment with the volume fraction of $v_{p}$. The model also incorporates blood plasma flow $F_{p}$ for the vascular transport of the contrast agent from the artery $\left(C_{a}(t)\right)$ to the capillary plasma compartment $\left(C_{p}(t)\right)$ where the contrast agents at the capillary bed are actively exchanged bidirectionally between the vascular and the EES with permeability surface area product PS. The contrast agent concentration of the tissue $\left(C_{t}(t)\right)$ can be described as the following:

[3] $$C_{t}(t)=v_{p}C_{p}(t)+v_{e}C_{e}(t)\;v_{p}\frac{dC_{p}}{dt}=F_{p}C_{a}(t)+PS\cdot C_{e}(t)-\left(F_{p}+PS\right)C_{p}(t)\;v_{e}\frac{dC_{e}}{dt}=PS\cdot C_{p}(t)-PS\cdot C_{e}(t)$$

where $C_{a}(t),C_{p}(t)$ and $C_{e}(t)$ are the contrast agent concentrations in the artery, in the capillary plasma and in the EES, respectively. Note that the exchange rate of $K^{trans}$ is related to $F_{p}$ and PS with the following equation:

[4] $$K^{\text{trans}}=F_{p}\cdot \left(1-exp\left(-\frac{PS}{F_{p}}\right)\right).$$

The arterial input function (AIF), denoted above as $C_{a}(t)$, was sampled from the aorta using Rocketship, a dedicated toolkit for AIF sampling^48^. For the purpose of comparing PK parameters across GRASP reconstruction methods, a population-based AIF was generated as an average for all malignant and benign cases, while keeping its original rise time per case. The pre-contrast T1 values were adopted from previous reports; 1.5 s/1.4 s for malignant / benign lesions^49^ and 1.3 s for fibroglandular tissue^50^. To improve the model fit to the signal, bootstrapping approach was used, where we randomly collected 10% of given voxels and averaged their contrast dynamics. Then the averaged contrast dynamics was fitted with our populated AIF. We repeated these 100 times to establish the statistics of PK parameters for each region. PKM analysis was applied across three distinct groups: fourteen malignant cases, twenty-one benign and twenty-one normal-appearing fibroglandular tissue.

## Supplementary Material

1

## Data Availability

In an effort to enhance accessibility and reproducibility, the raw radial k-space data used in this study will be available through our dedicated fastMRI repository^31^ (https://fastmri.med.nyu.edu/).
