# Supplementary material for: Dynamic MRI with Locally Low-Rank Subspace Constraint: Towards 1-Second Temporal Resolution Aided by Deep Learning
Source: Res Sq. 2025 Feb 27:rs.3.rs-5448452. Preprint. [Version 1] doi: 10.21203/rs.3.rs-5448452/v1 (PMC11888544; doi:10.21203/rs.3.rs-5448452/v1)
Supplement: 1 [file NIHPPRS5448452V1-supplement-1.pdf]

Supplementary material:

Supplementary Fig. 1: GLR and LLR reconstruction approaches for breast DCE-MRI. (a) Globally low-rank (GLR) estimates its basis function based on the whole image. (b) For locally low-rank (LLR), the image can be divided either into blocks ('LLR-Block') or (c) tissue-segments ('ELITE'), where each tissue can be represented by its own set of unique singular values decay pattern (far right).

Supplementary Fig. 2: Structural differences in breast tissue, reconstructed by GRASP methods. (a-e) Five representative cases where mild streak artifacts (red arrows) overlap and obscure fine breast structures. The same regions, reconstructed with the proposed ELITE framework, show clearer anatomy with minimal streak artifacts and similar appearance (yellow arrows), when compared to cartesian VIBE (acquired before contrast injection). Note that in the case of GRASP-LLR, some residual steak artifacts still appear due to blocks located at the boundary between tissue and background signal. All GRASP data were reconstructed based on early contrast phase.

Supplementary Fig. 3: Pharmacokinetic parameter maps of a representative healthy tissue case. Parameter maps overlaid on magnitude images calculated based on NUFFT, GRASP, GRASP-GLR, GRASP-LLR and ELITE reconstruction methods. Ve and Ktrans ranging [0 0.8], Vp [0 0.4] and Fp [0 1.5].

Supplementary Fig. 4: Pharmacokinetic parameter maps of a representative benign lesion case. For information see Supplementary Fig. 3.

Supplementary Fig. 5: Pharmacokinetic parameter maps of a representative malignant lesion case. For information see Supplementary Fig. 3.

Supplementary Fig. 6: Pre-, post- and sub-DCE images of a representative malignant case. Magnitude images were generated by NUFFT, GRASP, GRASP-GLR, GRASP-LLR and ELITE reconstruction methods. Intense streak artifacts are indicated by red arrows.

Supplementary Fig. 7: Pre-, post- and sub-DCE images of a representative malignant case. For information see Supplementary Fig. 6.

Supplementary Fig. 8: Pre-, post- and sub-DCE images of a representative malignant case. For information see Supplementary Fig. 6.

Supplementary Fig. 9: Pre-, post- and sub-DCE images of a representative malignant case. For information see Supplementary Fig. 6.

Supplementary Fig. 10: Pre-, post- and sub-DCE images of a representative benign case. For information see Supplementary Fig. 6.

Supplementary Fig. 11: Compressed-sensing reconstruction iterative reconstruction. Iterative reconstruction for GRASP-LLR and ELITE, based on Eq. 2, shows fewer number of iterations until convergence with stop criteria below 2.5%, compared to GRASP and GRASP-GLR.

Supplementary Fig. 12: Improving Fat saturation performance. (a) Reference anatomical image mainly consists of fat tissue. With ELITE, (b) fat tissue (blue mask) is represented with a single principal component, (c) resulting in a clear and sharper image with no streak artifacts, and with high correspondence to the conventional VIBE image.

Supplementary Fig. 13: ELITE: number of principal components (PC). (a) A representative malignant lesion case. (b) Signal enhancement ratio curve with six PC's achieves the highest temporal correlation and lowest norm (0.98/1.04, respectively), in comparison to NUFFT. (c) The lowest background noise during pre- and post-contrast is also achieved with six PC's.

Supplementary Fig. 14: ELITE: Lamba value. High-resolution iterative reconstruction with λ=0 shows some residual noise, specially at the margins of the skin and surrounding fibroglandular tissue, while higher regularization term (λ=0.1) results in slightly blurry tissue due to over-regularization.

Supplementary Video 1: Streak artifacts time-series experienced by the different GRASP reconstruction methods. Note the substantial reduction in background noise and streak artifact that is achieved with ELITE.

Supplementary Video 2: Streak artifacts time-series experienced by the different GRASP reconstruction methods. Note that in some cases GRASP-LLR may still show some residual streak artifacts, due to blocks located on the boundary between tissue and background signal.

Supplementary Video 3: Representative undersampled GRASP image-series as ‘input’ and ‘output’ of ResNet, reconstructed with a temporal resolution of 1-sec/2 spokes.

Supplementary Files

This is a list of supplementary files associated with this preprint. Click to download.

- Suppvideo1.avi
- Suppvideo2.avi
- Suppvideo3.avi
- supplementaryinformationfinal.docx
